# Preventing Substance Abuse in Adolescents: A Review of High-Impact Strategies

**DOI:** 10.7759/cureus.27361

**Published:** 2022-07-27

**Authors:** Hailey Hsiung, Karan Patel, Henna Hundal, Basil M Baccouche, Kuang-Wen Tsao

**Affiliations:** 1 Substance Abuse Prevention, Highland Park Municipal Alliance, Highland Park, USA; 2 Medical School, Cooper Medical School, Camden, USA; 3 Medical School, Stanford University School of Medicine, Stanford, USA; 4 Palliative Care, Vitas Healthcare, Middlebury, USA

**Keywords:** evidence-based, substance use prevention, blueprints, rat park, substance use disorder, opioids, youth, adolescents, substance abuse, prevention programs

## Abstract

Substance abuse has been an intractable societal concern in the US for more than half a century. The recent opioid epidemic has only accentuated this problem. Adolescents are significant long-term contributors to the crisis due to their susceptibilities to drug abuse and impressionable age. This review examines the particular vulnerabilities of the adolescent brain to drug abuse and the risk and protective factors thereof, especially in light of the Rat Park studies. In addition, the article provides an overview of the evidence-based prevention program registries and offers detailed summaries of two: Blueprints for Healthy Youth Development (Blueprints) and the Washington State Institute for Public Policy (WSIPP). By combining inputs from Blueprints and WSIPP, five programs with the highest benefit-cost ratios (BCR) were identified: Functional Family Therapy, Positive Family Support, Lifeskills Training, Positive Action, and Good Behavior Game. In light of their outstanding characteristics, these programs are poised to be widely implemented and to make a measurable difference in the fight against substance and opioid abuse.

## Introduction and background

Substance abuse and addiction is a protracted societal problem that has long defied attempts to tame it. Since the War on Drugs was declared more than 50 years ago, the situation has not improved. In fact, it has worsened with the recent opioid crisis. According to the National Institute on Drug Abuse, the cost of substance abuse in the United States, including that of healthcare, lost productivity, addiction treatment, and criminal justice involvement, is approximately $600 billion annually [1], with prescription opioid misuse accounting for $78.5 billion [2]. Adolescents are especially vulnerable to substance abuse. In 2020, people ages 15 to 24 experienced the greatest percentage increase in deaths due to drug overdose [3]. Despite the discouraging statistics, however, there has been significant and accelerating scientific progress toward the prevention and treatment of substance use disorder (SUD). The purpose of this paper is to review the literature and highlight the progress and new ideas in SUD prevention, especially as pertaining to adolescents.

## Review

The adolescent brain: susceptibility and vulnerability

Adolescence is a critical time for the development of the brain. During this period, which continues until the mid-twenties, cognitive and social skills develop, and the brain changes to prepare the teenager for the independence of adulthood [4,5]. Numerous studies have demonstrated that adolescents are especially susceptible to drug use compared to adults because of these neuroanatomical changes, including those occurring in the prefrontal cortex (PFC), striatal cortex, and limbic system. The PFC, which is the reasoning and decision-making part of the brain, grows during childhood but is pruned back during adolescence [5,6]. At the same time, the teenage striatal cortex becomes more sensitive to immediate rewards such as sugar and money, when compared to that of a child or adult [7]. Furthermore, the limbic region, which processes emotion and memory, matures earlier during adolescence, while the PFC lags behind and continues to develop until the age of 25 [8]. For these reasons, adolescents tend to make decisions based on emotion and immediate rewards instead of long-term consequences, making them more likely to experiment with drugs.

More concerningly, the developing adolescent brain is also more vulnerable to addiction and the damaging effects of substance abuse than the adult brain. In an experiment designed to model the effects of adolescent drug use, it was found that cocaine use altered the gene expression patterns and histone modification in the PFC of the rats, suggesting that cocaine exposure during adolescence has profound and long-lasting cellular and behavioral consequences even after the drug is no longer administered [9]. Human studies revealed equally troubling findings. In a recently published study that followed over 5000 people for 32 years, from ages 18 to 50, researchers found that, among individuals with severe SUD symptoms at 18, 62% still experienced two or more SUD symptoms in adulthood. In addition, they also had the highest adjusted odds of prescription drug use as adults. These findings suggest that individuals with severe SUD symptoms as adolescents do not grow out of their drug problems; they also face more severe long-term consequences than adolescents with no or low SUD symptoms [10].

Common socially and culturally tolerated substances affect the development of the brain. Alcohol, for example, can cause long-lasting neurophysiological changes, including alterations in both gray-matter and white-matter brain structures, as well as aberrations in brain activity. These structural and functional differences translate into poorer performances in neurocognitive tests of attention, working memory, spatial functioning, verbal and visual memory, and executive functioning [11]. Nicotine, another substance popular amongst teenagers, has been shown to negatively affect impulse control, attention span, memory, and executive function in adolescents. Compared to non-smokers, teenage smokers are significantly more likely to use other drugs, engage in high-risk sexual behavior, and develop psychiatric disorders. In addition, adolescents also experience greater pleasure than adults from nicotine due to their overdeveloped excitatory glutamatergic system, which facilitates dopaminergic neurotransmission, as well as underdeveloped inhibitory GABAergic system [12]. For this reason, the age of first cigarette use is a risk factor for nicotine dependence [13]. In fact, approximately 90% of adult smokers began smoking prior to turning 18 years of age [12], suggesting that adolescence is a critical developmental window related to lifelong nicotine dependence.

The adolescent brain is a dynamic and changing organ, second only to the infant brain in terms of synaptogenesis [5]. With its traits of sensation seeking and risk-taking, it is optimized for survival in the natural environment but is ill-prepared for the modern world, in which addictive substances are widely available. For one, it is prone to drug use. It is also especially vulnerable to the negative effects of drug use. These twin vulnerabilities are the reasons adolescents should be prime targets for substance use prevention.

New ideas in SUD prevention and treatment

One of the most influential new ideas in SUD research is actually more than 40 years old: In the late 1970s, Canadian researchers Bruce K. Alexander and colleagues housed rats either individually in small cages known as Skinner boxes or socially in a mixed-sex colony known as the Rat Park, which was 200 times larger than normal cages and offered a variety of compartments for play and social enrichment. The experiments showed that, while the socially isolated rats obsessively self-administered morphine until they died, the Rat Park rats mostly abstained from morphine water; they would try it, but not to the point of addiction and overdose. In fact, they showed a statistically greater preference for plain water over morphine water [14,15]. In another experiment, Alexander and colleagues forced rats to become addicted by giving them only morphine-laced solutions for over 50 days. When these rats were moved into Rat Park, they chose to drink plain water instead of the morphine solution and showed minimal signs of withdrawal and dependence [15,16]. This is a significant finding, especially in the context of the War on Drugs, which focused almost exclusively on the largely failed approach of prohibition and supply reduction [17-19]. These studies pointed to a way to actually reduce the demand for drugs. With insights gained from his Rat Park studies, Alexander proposed that SUD should be considered a manifestation of social isolation and dislocation [20].

Alexander’s landmark studies were ignored for more than 30 years. However, they have enjoyed renewed research interests in the last decade. In a recent study, researchers revisited the Rat Park experiments, but with a twist: Instead of choosing between drugs and no drugs, the rats had to choose between drugs and social interaction. In this scenario, the rats pressed the lever to enter the “social peer chamber” instead of the “drug self-administration chamber” more than 90% of the time - even for the rats that had previously been exposed to methamphetamine for three weeks and exhibited signs of addictive behavior. This finding corroborated with Alexander’s finding that addicted rats in Rat Park preferred plain water to morphine. The research also highlighted a qualitative difference in rat addiction behaviors, initially identified by Alexander, between the voluntary abstinence rats that chose social reward and the involuntary abstinence rats that had their drugs removed - with the former showing little or no signs of drug craving behavior while the latter showing an intensification of drug craving behaviors over time known as the “incubation of craving” [21]. This suggests that social reward has a protective effect on drug-addicted rats by alleviating the expected withdrawal symptoms. Further experiments pinpointed this protective effect as due to the inhibition of the activities in the central amygdala and the anterior ventral insular cortex, which are brain regions related to drug craving [21].

Studies in humans also support the link between social factors and SUDs. For example, negative experiences such as bullying, social conflict, and economic stress are found to be common triggers of drug relapse. On the other hand, positive social experiences, such as having friends and social support, can be restorative factors over relapse [22-25]. Therefore, it is not surprising that therapies that improve the adolescents' most important social environments - their families - are found to be helpful in the treatment of SUDs. A meta-analysis by Tanner-Smith et al. found family therapy programs to be more effective than other therapy programs, such as behavioral therapy, cognitive behavioral therapy, motivation enhancement therapy, psychoeducational therapy, group counseling, and “practice as usual” (the default therapies that served as controls). Their study revealed that family therapy programs resulted in a 40% greater reduction in drug use than did other treatments [26].

Another well-supported evidence-based drug treatment method, community reinforcement approach (CRA), also takes advantage of the therapeutic benefits of positive social interactions. It focuses on helping clients to become more positively engaged with their families, friends, school, work, and community organizations and to enhance the enjoyment and frequency of non-drug-related social activities. A meta-analysis of six outcome studies shows CRA’s effect size for SUD to be large (ES=-0.58) and highly significant [26,27].

Risk and protective factors of SUD

Over the years, addiction researchers have identified many factors associated with SUD. Some are risk factors that make an individual more prone to develop the disorder while others are protective factors that make an individual less likely to do so [28,29]. In light of the importance of the social factor in SUD prevention and treatment, as revealed by the Rat Park and more recent studies, many of the risk and protective factors may be broadly categorized as factors that weaken or strengthen social ties. The remaining risk and protective factors may be categorized as factors that are either restrictive or permissive toward drug use. As shown in Table 1, we categorized “Poor control over school drug consumption” and “Availability and cost of drugs and alcohol” as risk factors related to the category of “Permissiveness (toward drug use).” On the other hand, “Opportunity for fulfilling extracurricular activities” and “Attachment or sense of belonging to school” were considered protective factors in the “Social ties” category. We also grouped “Psychiatric disorder” and “Emotional distress” as individual risk factors related to “Social ties” since they interfere with normal social functioning.

**Table 1:** Substance misuse risk and protective factors Adapted and modified from JAMA Psychiatry [29] and US Department of Health and Human Services [30].

	Social ties		Permissiveness	
	Risk Factors	Protective factors	Risk Factors	Protective factors
Individual	Aggressiveness that starts early and is persistent	Interpersonal skills: social, emotional, and cognitive	Starting substance use early	Delayed substance use into late adolescence or early adulthood
	Psychiatric disorder	Treatment of psychiatric disorders	Perceiving little risk in substance use	Drug resistance skills
	Emotional distress	Resiliency, self-efficacy, and spirituality		
Family	Family conflict, abuse, or neglect	Strong connection with immediate and extended family	Substance misuse in the family	Parents who do not misuse drugs and do not approve of substance use
		Meaningful involvement with family	Parents who favorably view or approve of substance use	
		Positive behavior is recognized by parents and caretakers		
		Being in a committed relationship or marriage with a partner who does not misuse drugs		
School	Poor academic performance	Academic competence	Peers who use substances	School norms that drug misuse is not acceptable
	Lacking commitment to school and not viewing school as rewarding or meaningful	Attachment or sense of belonging to school	Poor control over school drug consumption	
		Positive behavior is recognized by teachers	Perception that the use of drugs among classmates is high	Accurate perception of drug use prevalence among classmates
		Opportunity for fulfilling extracurricular activities		
Community	Lower socioeconomic status	Attachment or sense of belonging to community, culture, or ethnic group	Availability and cost of drugs and alcohol	Norms in community that drug use is not acceptable
		Meaningful involvement with community	Community norms favorable toward alcohol and drugs	
		Positive behavior is recognized in community		

Another way to look at the two categories in Table 1 is in terms of supply and demand. In the “Permissiveness” category, the risk factors are related to increases in actual or perceived drug supply, while protective factors are related to reductions in actual or perceived drug supply. In the “social ties” category, the risk factors are related to increases in the demand for substance consumption, while the protective factors are related to decreases in the demand for drug consumption. For example, individuals suffering from anxiety and depression or living in high-conflict families have a higher tendency to consume drugs, while resilient individuals with close-knit families have a lower tendency to do so.

Risk and protective factors are correlated and cumulative over time [28]. For example, parental substance misuse is associated with dysfunctional parenting and emotional trauma, which in turn can lead to poor academic performance and lower socioeconomic status. For this reason, prevention efforts targeting a particular risk or protective factor often lead to positive outcomes in multiple areas. All the effective SUD evidence-based prevention programs (EBPP) produce multiple positive outcomes, ranging from improved academic performance to reduced violent behaviors - in addition to reductions in substance use. Therefore, unlike prescription medications, most of the “side effects” of EBPPs are actually positive.

Evidence-based prevention program registries

EBPPs work by reducing risk factors of SUD or strengthening protective factors [30,31]. The National Academy of Medicine classifies EBPPs according to their targeted population; it identifies three overlapping categories based on their level of risk: Universal, Selective, and Indicated. With respect to SUD prevention, universal programs target all members of a population - for instance, all students in the school district; selective programs are aimed at high-risk subgroups such as individuals with personality or hereditary traits that predispose them to SUD; indicated programs aimed at early substances users who have not yet developed SUDs [32,33].

In the past 30 years, there has been an explosion in the development and evaluation of EBPPs. In response to this flood of data, a number of EBPP registries have emerged to evaluate, catalog, and rank these programs, as shown in Table 2.

**Table 2:** Evidence-based substance use disorder (SUD) prevention program registries EBPP: evidence-based prevention programs

EBPP Registries
Blueprints for Healthy Youth Development (Blueprints)
Washington State Institute for Public Policy (WSIPP)
Crime Solutions of the National Institute of Justice
Model Programs Guide of the Office of Juvenile Justice and Delinquency Prevention
What Works Clearinghouse of the Institute of Education Sciences at the U.S. Department of Education
Evidence-Based Practices Resource Center of the Substance Abuse and Mental Health Services Administration
Youth.gov Program Directory
California Evidence-Based Clearinghouse
“Preventing Drug Use among Children and Adolescents: A Research-Based Guide for Parents, Educators, and Community Leaders” Second Edition, by National Institute on Drug Abuse (NIDA)

Of these programs, Blueprints and WSIPP deserve special mention.

Blueprints, founded in 1996, was one of the earliest efforts to evaluate EBPPs according to a clear set of scientific standards and a rigorous expert review process. It has been recognized by practitioners and researchers as a premier registry for EBPPs for adolescents [34,35] and is arguably the most user-friendly information portal for decision-makers and program implementers. For each Blueprints certified program, it provides a “fact sheet” that details the basic information about the program, including program description, program outcomes, and endorsements by other registries. It also provides information about program costs, training, technical assistance, and funding strategies [36].

Blueprints certified programs are rated as Promising, Model, or Model Plus. The Promising programs must meet a minimum standard of effectiveness and require either one randomized control trial or two quasi-experimental trials. The Model programs are programs whose results are replicated by additional randomized and/or quasi-experimental trials. The highest-tier Model Plus programs must meet Blueprints’ most rigorous scientific evaluation and require the program outcome to be independently replicated by researchers who are not affiliated with the program developer [36]. Additionally, all three program types must have the necessary organizational capability, manuals, training, technical assistance, and other supporting infrastructure required for high-quality implementation in communities and schools. Blueprints programs, therefore, are not only evidence-based but also implementation ready [34].

WSIPP takes a unique approach to EBPP evaluation. In addition to reviewing the research literature to identify effective EBPPs, it also estimates their economic benefits, providing policymakers with the requisite numbers to make policy or funding decisions. The WSIPP benefit-cost model does this by valuing changes in outcomes produced by the programs and comparing them to the costs of providing those programs. The benefit and cost estimates reflect the difference between a person who participates in the program and one who does not. Finally, WSIPP runs a sensitivity analysis, known as Monte Carlo analysis, to account for the risk and uncertainty around many of the inputs and assumptions of the model. As a part of this analysis, the model calculates the benefit-cost results of an EBPP 10,000 different times, each time varying the inputs randomly within a defined range. The output of this calculation - “the chance the program will produce benefits greater than the costs” - reflects the percentage of those runs in which the benefits minus the costs are greater than zero [37]. Therefore, for each EBPP, the reader has two useful numbers to work with: What is the estimated return on investment of the program and how risky is this estimate (i.e., the probability that the program will at least break even) [38].

It should be noted that the unit of measurement of benefit-cost analysis is the “value of a statistical life” (VSL). This means that the benefits calculated are limited to the lifetime of the intervention recipients [39]. However, research now demonstrates that EBPPs yield benefits beyond the lifetime of the intervention recipients - to the next generation. In the first study of its kind, a long-term follow-up of the Raising Healthy Children program found that the children of the original participants, who are now parents, demonstrated better academic skills, fewer behavior problems, and lower incidence of SUD [40]. If additional cross-generational studies bear fruit, then the WSIPP model may well underestimate the very long-term benefits of EBPPs.

Blueprints and WSIPP recommended EBPPs

With Blueprints [41] providing rigorous evaluations of efficacy and WSIPP [42] providing the cost-benefit analysis, we have the tools to quickly identify the most promising EBPPs that are implementation ready and have a high probability of success. Table 3 lists all of the Blueprints certified programs related to substance use prevention, with the two benefit-cost analysis numbers drawn from the table on the WSIPP website. The two numbers are the Benefit-Cost Ratio (BCR) and the chance benefits will exceed costs (Predictability).

**Table 3:** Blueprints certified substance use prevention programs with benefit-cost ratios Adapted and modified from Blueprints [41] and WSIPP [42].

Program Name	Blueprints Rating	Benefit-to-cost ratio	Predictability (chance benefits will exceed costs)	Setting	Continuum of Intervention	Target Population	Outcomes
Functional Family Therapy (FFT)	Model Plus	$ 18.75	100%	Mental Health/Treatment Center	Selective/ Indicated Prevention	Age: Late Adolescence (15-18) - High School, Early Adolescence (12-14) - Middle School Race / Ethnicity: All Gender: Both	Delinquency and Criminal Behavior, Externalizing, Illicit Drugs
LifeSkills Training (LST)	Model Plus	$ 13.49	63%	School	Universal Prevention	Age: Early Adolescence (12-14) - Middle School Race / Ethnicity: All Gender: Both	Alcohol, Delinquency and Criminal Behavior, Illicit Drugs, Sexual Risk Behaviors, STIs, Tobacco, Violence
Multisystemic Therapy® (MST®)	Model Plus	$ 3.02	99%	Mental Health/Treatment Center	Indicated Prevention	Age: Late Adolescence (15-18) - High School, Early Adolescence (12-14) - Middle School Race / Ethnicity: All Gender: Both	Close Relationships with Parents, Conduct Problems, Delinquency and Criminal Behavior, Externalizing, Illicit Drugs, Internalizing, Mental Health - Other, Positive Social/Prosocial Behavior, Prosocial with Peers, Violence
Blues Program	Model	$ (0.44)	49%	School	Selective/ Indicated Prevention	Age: Late Adolescence (15-18) - High School Race / Ethnicity: All Gender: Both	Depression, Illicit Drugs
Brief Alcohol Screening and Intervention for College Students (BASICS)	Model	$ 12.49	66%	School	Selected/ Indicated Prevention	Age: Early Adulthood (19-22) Race / Ethnicity: All Gender: Both	Alcohol
Multisystemic Therapy Problem Sexual Behavior (MST-PSB)	Model	$ 1.55	59%	Mental Health/Treatment Center	Indicated Prevention	Age: Late Adolescence (15-18) - High School, Early Adolescence (12-14) - Middle School Race / Ethnicity: All Gender: Both	Academic Performance, Adult Crime, Delinquency and Criminal Behavior, Illicit Drugs, Mental Health - Other, Prosocial with Peers, Sexual Risk Behaviors, Sexual Violence
Positive Action	Model	$ 29.32	94%	School	Universal Prevention	Age: Early Adolescence (12-14) - Middle School, Late Childhood (5-11) - K/Elementary Race / Ethnicity: All Gender: Both	Academic Performance, Alcohol, Anxiety, Bullying, Close Relationships with Peers, Delinquency and Criminal Behavior, Depression, Emotional Regulation, Illicit Drugs, Positive Social/Prosocial Behavior, Sexual Risk Behaviors, Tobacco, Truancy - School Attendance, Violence
Project Towards No Drug Abuse	Model	$ 5.71	54%	School	Universal/ Selective Prevention	Age: Late Adolescence (15-18) - High School Race / Ethnicity: All Gender: Both	Alcohol, Illicit Drugs, Tobacco, Violence
Treatment Foster Care Oregon	Model	$ 4.29	90%	Community	Indicated Prevention	Age: Late Adolescence (15-18) - High School, Early Adolescence (12-14) - Middle School Race / Ethnicity: All Gender: Both	Delinquency and Criminal Behavior, Illicit Drugs, Teen Pregnancy, Tobacco, Violence
A Stop Smoking in Schools Trial (ASSIST)	Promising	n/a	n/a	School	Universal Prevention	Age: Early Adolescence (12-14) - Middle School Race / Ethnicity: All Gender: Both	Tobacco
Achievement Mentoring	Promising	n/a	n/a	School	Selective Prevention	Age: Early Adolescence (12-14) - Middle School Race / Ethnicity: All Gender: Both	Academic Performance, Delinquency and Criminal Behavior, Employment, Illicit Drugs, Truancy - School Attendance
Athletes Training and Learning to Avoid Steroids (ATLAS)	Promising	n/a	n/a	School	Universal Prevention	Age: Late Adolescence (15-18) - High School Race / Ethnicity: White Gender: Male	Alcohol, Illicit Drugs, Physical Health and Well-Being
Big Brothers Big Sisters of America	Promising	$ (0.06)	4%	Community	Selective Prevention	Age: Late Adolescence (15-18) - High School, Early Adolescence (12-14) - Middle School, Late Childhood (5-11) - K/Elementary Race / Ethnicity: All Gender: Both	Alcohol, Antisocial-aggressive Behavior, Close Relationships with Parents, Close Relationships with Peers, Illicit Drugs, Positive Social/Prosocial Behavior, Truancy - School Attendance
Cannabis eCHECKUP TO GO	Promising	n/a	n/a	Online	Indicated Prevention	Age: Early Adulthood (19-22) Race / Ethnicity: All Gender: Both	Marijuana/Cannabis
Communities That Care	Promising	$ 5.20	86%	Community	Universal Prevention	Age: Early Childhood (3-4) - Preschool, Infant (0-2), Early Adulthood (19-22), Late Adolescence (15-18) - High School, Early Adolescence (12-14) - Middle School, Late Childhood (5-11) - K/Elementary Race / Ethnicity: All Gender: Both	Alcohol, Antisocial-aggressive Behavior, Delinquency and Criminal Behavior, Illicit Drugs, Tobacco, Violence
Coping Power	Promising	$ 1.25	55%	School	Selective Prevention	Age: Late Childhood (5-11) - K/Elementary Race / Ethnicity: All Gender: Both	Academic Performance, Alcohol, Antisocial-aggressive Behavior, Conduct Problems, Delinquency and Criminal Behavior, Illicit Drugs, Positive Social/Prosocial Behavior, Prosocial with Peers
EFFEKT	Promising	n/a	n/a	Community	Universal Prevention	Age: Early Adolescence (12-14) - Middle School Race / Ethnicity: All Gender: Both	Alcohol, Delinquency and Criminal Behavior
Familias Unidas	Promising	$ 3.50	68%	Community	Selective Prevention	Age: Late Adolescence (15-18) - High School, Early Adolescence (12-14) - Middle School Race / Ethnicity: Hispanic or Latino Gender: Both	Externalizing, Illicit Drugs, Sexual Risk Behaviors
Good Behavior Game	Promising	$ 62.80	76%	School	Universal Prevention	Age: Late Childhood (5-11) - K/Elementary Race / Ethnicity: All Gender: Both	Alcohol, Antisocial-aggressive Behavior, Illicit Drugs, Internalizing, Mental Health - Other, Suicide/Suicidal Thoughts, Tobacco
Guiding Good Choices	Promising	$1.36	50%	Community	Universal Prevention	Age: Early Adolescence (12-14) - Middle School Race / Ethnicity: All Gender: Both	Alcohol, Delinquency and Criminal Behavior, Depression, Illicit Drugs
InShape Prevention Plus Wellness	Promising	$ 1.41	49%	School	Universal Prevention	Age: Early Adulthood (19-22) Race / Ethnicity: All Gender: Both	Alcohol, Illicit Drugs
KEEP SAFE	Promising	n/a	n/a	Social Services	Selective Prevention	Age: Early Adolescence (12-14) - Middle School Race / Ethnicity: All Gender: Female	Illicit Drugs, Positive Social/Prosocial Behavior, Sexual Risk Behaviors, Tobacco
Learning Together	Promising	n/a	n/a	School	Universal Prevention	Age: Early Adolescence (12-14) - Middle School Race / Ethnicity: All Gender: Both	Alcohol, Bullying, Conduct Problems, Delinquency and Criminal Behavior, Illicit Drugs, Mental Health - Other, Tobacco
Positive Family Support	Promising	$ 197.66	70%	School	Universal/ Selective/ Indicated Prevention	Age: Early Adolescence (12-14) - Middle School Race / Ethnicity: All Gender: Both	Alcohol, Depression, Sexual Risk Behaviors, Tobacco
Project Northland	Promising	$ 2.73	54%	School	Universal Prevention	Age: Late Adolescence (15-18) - High School, Early Adolescence (12-14) - Middle School Race / Ethnicity: All Gender: Both	Alcohol
PROSPER	Promising	$ 0.76	39%	Community	Universal Prevention	Age: Early Adolescence (12-14) - Middle School Race / Ethnicity: All Gender: Both	Alcohol, Close Relationships with Parents, Conduct Problems, Delinquency and Criminal Behavior, Illicit Drugs, Tobacco
Raising Healthy Children	Promising	n/a	n/a	School	Universal Prevention	Age: Late Adolescence (15-18) - High School, Early Adolescence (12-14) - Middle School, Late Childhood (5-11) - K/Elementary Race / Ethnicity: All Gender: Both	Academic Performance, Alcohol, Antisocial-aggressive Behavior, Illicit Drugs, Prosocial with Peers
SPORT Prevention Plus Wellness	Promising	$ 5.81	51%	School	Universal Prevention	Age: Late Adolescence (15-18) - High School Race / Ethnicity: All Gender: Both	Alcohol, Illicit Drugs, Physical Health and Well-Being, Tobacco
Strengthening Families 10-14	Promising	$ 5.36	60%	Community	Universal / Selective Prevention	Age: Early Adolescence (12-14) - Middle School, Late Childhood (5-11) - K/Elementary Race / Ethnicity: All Gender: Both	Alcohol, Antisocial-aggressive Behavior, Close Relationships with Parents, Illicit Drugs, Internalizing, Tobacco
Strong African American Families -- Teen	Promising	$ 3.04	59%	Community	Universal Prevention	Age: Late Adolescence (15-18) - High School Race / Ethnicity: African American Gender: Both	Alcohol, Conduct Problems, Depression, Illicit Drugs, Sexual Risk Behaviors
Strong African American Families Program	Promising	$1.95	54%	Community	Universal Prevention	Age: Early Adolescence (12-14) - Middle School, Late Childhood (5-11) - K/Elementary Race / Ethnicity: African American Gender: Both	Alcohol, Close Relationships with Parents, Delinquency and Criminal Behavior, Truancy - School Attendance

All three Model Plus programs have positive BCRs, and two of the three have outstanding numbers: Every dollar of investment yields $19 in benefits from the Functional Family Therapy (FFT) program and $13 in benefits from LifeSkills Training (LST). Among Model Programs, Positive Action (PA) stands out with a BCR of $29. It also has the most wide-ranging positive outcomes, from improved academic performance to reduced bullying, to reduced substance abuse. Among Promising programs, Good Behavior Game (GBG) and Positive Family Support (PFS) stand out with returns of more than $63 and $198 for every dollar invested - the two highest BCRs amongst all Blueprints-certified programs related to SUD. Except for FFT (a selective/indicated program) and PFS (a hybrid of all three types), all programs are universal interventions delivered in school settings. Below is a brief description of each program:

Functional Family Therapy (FFT)

FFT is a selective EBPP targeting at-risk adolescents who have been referred by juvenile justice systems, healthcare providers, child welfare agencies, or schools. It is a strength-based short-term family counseling program that is provided primarily in clinical settings but may also be provided at home, schools, child welfare agencies, and probation and parole systems. At 100%, it has the highest predictability rating amongst all Blueprints programs, meaning that it is a near certainty that benefits will exceed costs and that the program will succeed [43].

Lifeskills Training (LST)

Developed by Dr. Gil Botvin at Cornell University, LST is a three-year universal substance abuse and violence EBPP designed to be implemented with middle school students. It consists of 15 core sessions in Level 1, 10 booster sessions in Level 2, and five booster sessions in Level 3. Additional topic-specific supplemental lessons (targeting opioid or violence prevention, for instance) also are available for each level. Units are taught sequentially and delivered primarily by classroom teachers. LST provides students with training in personal self-management, social skills, and drug resistance skills. Skills are taught using instruction, demonstration, feedback, reinforcement, and practice [32]. It has a predictability factor of 63%, meaning that there is a 63% chance that the benefits of the implemented program will exceed the costs.

Positive Action (PA)

PA was developed by Carol Gerber Allred, Ph.D., a public school teacher and administrator, more than 30 years ago. The program is based on the intuitive philosophy that positive actions lead to positive feelings, leading to more positive actions. It consists of seven units, and every grade level is taught the same lessons but in an age-appropriate manner. The seven units are self-concept, mind and body, self-management, social conduct, self-honesty, self-improvement, and review. Supplemental modules help educators integrate other topics into the program such as SUD and bullying prevention [44]. PA has the highest BCR among Model and Model Plus programs, and the third-highest among all Blueprints programs, just after GBG and PFS. The lessons are short, interactive, scripted, and are designed to be delivered by the teacher with minimum preparation; this may account for its predictability score of 94%, which is unusually high for a universal intervention program.

Good Behavior Game (GBG)

First described and tested in 1969, GBG is a universal intervention originally designed as a classroom management strategy that rewards children for appropriate behavior during instructional times. The class is divided into two teams; points are given to the team with inappropriate behavior displayed by any of its members. Like golf, the team with the lowest score wins [45]. Even though GBG is not a Model or Model Plus Blueprints program, it (along with PFS) is one of the two programs in the Promising category with the highest potential. It has a BCR of 63 and a predictability score of 76%. Furthermore, in the well-respected list of EBPPs showcased in NIDA’s second edition of “Preventing Drug Use Among Children and Adolescents,” GBG was a part of three different multi-component EBPPs: Classroom Centered Intervention, Linking the Interests of Families and Teachers, and PROmoting School-community-university Partnerships to Enhance Resilience (PROSPER).

Positive Family Support (PFS)

PFS is an EBPP targeted toward at-risk middle school-aged children. Its goal is to reduce problem behavior and drug use by improving the parents' family management and communication skills and by addressing dysfunctional family dynamics. PFS is the only program in Table 3 that covers all three levels of intervention. At the universal level, information is provided to parents through books and videotapes. The goal is to establish positive parenting practices and to inform parents about risks that can lead to problem behavior and substance use. At the selective level, the program includes interactive interventions, such as Family Check-Up: three short family intervention sessions with a therapist consisting of an initial interview, an assessment session, and a feedback session. At the indicated level, direct professional support, such as behavioral family therapy, monitoring systems for academic and social behavior, parenting groups, referral services, and case management services are provided [46]. Studies have shown that PFS is effective in improving family interactions and reducing antisocial behavior and drug use [47]. This program’s success is backed up by its numbers: in addition to having a 70% predictability score, PFS has a $198 BCR - the highest in Table 3.

It is also interesting to note that, of the three highly rated universal, school-based programs listed above (LST, PA, and GBG), only LST was designed specifically to be a SUD EBPP. The other two, PA and GBG, had other intents but nonetheless had the effect of reducing substance use as one of their outcomes, a likely result of the Rat Park Effect - that social and environmental enrichments in themselves reduce substance abuse and addiction. The GBG program, for example, is most strongly indicated for aggressive male elementary school students, who, as a result of the intervention, exhibited significantly less aggressive and disruptive behavior than their control classroom counterparts. This reduction in aggression yielded subsequent long-term benefits; by the time they reached young adulthood, the formerly aggressive GBG kids were less likely to develop SUDs (29% vs 83% in controls) and less likely to display violent and criminal behavior (34% vs 50%) [45]. By reducing the aggressive and disruptive behavior, it appears that GBG had the effect of rescuing the aggressive students out of highly negative social environments created by their own aggressiveness and placed them into more accepting and amiable social environments - the human equivalent of moving out of Skinner boxes into Rat Park.

## Conclusions

Substance use amongst adolescents has long been a serious public health concern. To address this issue, decades were spent on the development and testing of substance use EBPPs. We finally now have effective, proven, and economically-sensible prevention programs. According to WSIPP, the net program cost of LST was $105 per student. As such, it costs just $5 billion dollars to roll out the highest-rated Blueprints program to all 50 million students in the country and generate societal benefits of more than $65 billion dollars. Therefore, at minimal costs, school-based universal interventions such as LST, PA, and GBG have the potential to transform our schools, turning environments of academic stress, bullying, and social exclusion, where adolescents first encountered and are hooked on drugs, into spaces where practical life and social skills are mastered. At the same time, effective family-focused programs such as PFS and FFT reduce familial dysfunction and improve the communication and relationship between the parent and child. Given that the Rat Park experiments and more recent studies have suggested that substance use may be related to the absence of fulfilling relationships, improving familial bonds and peer relationships work to effectively diminish the attraction of substances ranging from alcohol to opioids. A sense of urgency is needed to muster the necessary resources and implement these valuable programs in schools across the country.
